# Trimethylamine N-Oxide Exacerbates Cardiac Fibrosis via Activating the NLRP3 Inflammasome

**DOI:** 10.3389/fphys.2019.00866

**Published:** 2019-07-09

**Authors:** Xueling Li, Jin Geng, Jinxuan Zhao, Qianqian Ni, Chenze Zhao, Yaru Zheng, Xiaomin Chen, Lihong Wang

**Affiliations:** ^1^Department of Cardiology, Zhejiang Provincial People’s Hospital, People’s Hospital of Hangzhou Medical College, Hangzhou, China; ^2^Department of Cardiology, Huai’an First People’s Hospital, Nanjing Medical University, Huai’an, China; ^3^Department of Cardiology, Drum Tower Hospital, Nanjing University Medical School, Nanjing, China; ^4^Department of Medical Imaging, Jinling Hospital, Medical School of Nanjing University, Nanjing, China

**Keywords:** cardiac fibrosis, TMAO, NLRP3 inflammasome, cardiac fibroblast, doxorubicin

## Abstract

**Background/Aims:** Gut microbiota has been reported to correlate with a higher mortality and worse prognosis of cardiovascular diseases. Trimethylamine N-oxide (TMAO) is a gut microbiota-dependent metabolite of specific dietary nutrients, which is linked to cardiac fibrosis. Recent reports have suggested that the activation of Nucleotide-binding oligomerization domain (NOD)-like receptor protein 3 (NLRP3) inflammasome contributed to cardiac fibrosis. However, whether TMAO mediates cardiac fibrosis via activating NLRP3 inflammasome remains unclear.

**Methods and Results:** To determine the role of TMAO–mediated cardiac fibrosis, we established mouse models of doxorubicin (DOX)-induced cardiac fibrosis with or without TMAO in drinking water. TMAO exacerbated DOX-induced cardiac dysfunction, heart weight and cardiac fibrosis manifested by enhanced collagen accumulation, higher profibrotic levels and elevated inflammatory factors as well as NLRP3 inflammasome activation. Using primary cultured mouse cardiac fibroblast, our results indicated that TMAO promoted proliferation, migration and collagen secretion in a dose-dependent manner by TGF-β/Smad3 signaling. Furthermore, TMAO treatment induced NLRP3 inflammasome activation including oxidative stress in cultured cardiac fibroblast. Importantly, the silencing of NLRP3 presented a protection effect against cardiac fibrosis including cellular proliferation, migration and collagen deposition *in vitro*.

**Conclusion:** Our data suggested that TMAO aggravated DOX-induced mouse cardiac fibrosis, at least in part, through activation of the NLRP3 inflammasome, providing a new potential target for preventing the progression of cardiac fibrosis.

## Introduction

Cardiac fibrosis is characterized by excessive proliferation of cardiac fibroblasts and accumulation of extracellular matrix (ECM), which ultimately leads to cardiac diastolic dysfunction and heart failure (Creemers and Pinto, 2011; Travers et al., 2016). Cardiac fibroblasts are the predominant cell type located within interstitial tissues, contributing to the secretion of ECM proteins and influencing cardiac function in the presence of certain profibrotic stimuli (Lighthouse and Small, 2016; Xiang et al., 2017). Therefore, better strategies for regulating cardiac fibroblasts are essential for the development of effective anti-fibrosis intervention and delaying heart failure.

Trimethylamine N-oxide (TMAO) is a gut microbiota-dependent metabolite of specific dietary nutrients, including phosphatidylcholine, choline and carnitine (Wang et al., 2011). Previous studies have suggested that elevated plasma TMAO levels are associated with poor prognosis and increased mortality risk in cardiovascular disease, including heart failure (Tang et al., 2013; Heianza et al., 2017). Some clinical studies have reported TMAO levels to be higher with advanced left ventricular diastolic dysfunction in heart failure than in non-heart failure (Cannon and McMurray, 2014; Tang et al., 2014; Tang et al., 2015b). Several animal studies have suggested that elevated TMAO levels have a strong association with cardiac fibrosis and contribute to heart failure (Chen K. et al., 2017; Zhang et al., 2017; Li et al., 2018). However, the roles and mechanisms of TMAO-mediated cardiac fibrosis are not fully elucidated.

Nucleotide-binding oligomerization domain (NOD)-like receptor protein 3 (NLRP3), a member of the inflammasome family, could promote collagen production and cytokine interleukin (IL)-1β secretion, leading to fibrosis development in organs including the lung, liver and kidney (Song et al., 2016; Alegre et al., 2017; Wang et al., 2017). Recent evidence has shown that high glucose could promote NLRP3 inflammasome-mediated collagen synthesis, leading to caspase 1 activation and IL-1β secretion, which ultimately contribute to cardiac fibrosis in diabetic cardiomyopathy (Zhang et al., 2018). However, the role of the NLRP3 inflammasome in TMAO-mediated cardiac fibrosis remains unclear.

In the present study, we first examined the role of TMAO in doxorubicin (DOX)-induced cardiac fibrosis. Investigating the effects and exploring the underlying mechanisms of TMAO in primary cultured cardiac fibroblasts could provide novel therapeutic strategies based on NLRP3 inflammasome activation.

## Materials and Methods

### Animals and Treatments

C57BL-6J wild-type mice (6–8 weeks old males) were obtained from the Model Animal Research Center of Nanjing University (Nanjing, China). All mouse protocols were approved by the Institutional Ethics Committee of Nanjing Drum Tower Hospital and carried out according to the guidelines of the United States Department of Health (NIH Publication No. 85-23, revised 1996) for the use and care of laboratory animals. TMAO was purchased from Sigma (MO, United States). All mice were assigned to four groups (*n* = 10 per group): Control, TMAO, doxorubicin (DOX) or DOX+TMAO. To investigate the effects of TMAO on DOX-induced cardiac fibrosis, mice were intraperitoneally injected with four doses of DOX (6 mg/kg) or PBS every 3 days in accordance with a previous study (Rasanen et al., 2016). Moreover, all mice were treated with or without TMAO (120 mg/kg, 1.60 mmol/l) in drinking water as described previously (Makrecka-Kuka et al., 2017; Zhang et al., 2017). At 8 weeks after treatment, a mouse ultrasonic cardiogram was performed, and parameters including left ventricular end-systolic diameter (LVESD), left ventricular end-diastolic diameter (LVEDD), fractional shortening (FS) and ejection fraction (EF) were measured and analyzed with a Vevo2100 High-Resolution Micro-Ultrasound System (Visual Sonics, Toronto, Canada) (Li et al., 2017). Subsequently, the animals were sacrificed, and hearts and blood samples were collected.

### Histological Analysis

Heart tissues were fixed in 4% paraformaldehyde embedded in paraffin and sectioned at 5 μm intervals. Masson’s trichrome and immunohistochemical staining of collagen type I, NLRP3 and caspase 1 (1:200, Abcam, United States) were performed to analyze cardiac fibrosis using standard procedures as previously described (Yuan et al., 2017). Moreover, heart weight/body weight (HW/BW) and heart weight/tibia length (HW/TL) were also measured.

### Primary Mouse Cardiac Fibroblast Cultures and Treatments

Newborn ICR mice (1–2 days old) were anesthetized and sacrificed. Hearts were removed, and the ventricles were quickly and finely minced and digested with Trypsin-EDTA 0.125% (Invitrogen) and collagenase (Invitrogen, 1 mg/ml in DMEM) as we have described (Li et al., 2017). Subsequent supernatants were collected and centrifuged at 200 × *g* for 5 min and cultured in DMEM containing 4.5 g/L glucose plus 10% fetal bovine serum (FBS) and 1% penicillin/streptomycin (GIBCO) at 37°C. After 1.5 h, non-adherent and weakly attached cells were considered cardiac fibroblasts and cultured in new culture flasks. Cardiac fibroblasts after 2–3 passages were used in experiments (Piccoli et al., 2017; Liang et al., 2018). To evaluate the effect of TMAO on cultured cardiac fibroblasts, cardiac fibroblasts were treated with TMAO at different concentrations (10, 50, 100 μmol/l). The control groups were administered PBS alone.

### Small Interfering RNA Transfection and Treatments

SiRNA targeting mouse NLRP3 (5′-CAGCCAGAGTGGAATGACACGTGTA-3′) and control siRNA (5′-UUCUCCGAACGUGUCACG-3′) were synthesized by Genepharm Biotech (Shanghai, China). The second generation of cardiac fibroblasts was transfected with Lipofectamine 2000 (Invitrogen, Germany) according to the manufacturer’s instructions. At 48 h after transfection, cardiac fibroblasts were treated with AngII (100 nmol/l, Sigma, United States) or TMAO (100 μmol/l) or PBS as a control. The siRNA efficacy data was shown in Supplementary Figure S1 in Supplementary Materials.

### Western Blotting

Collected hearts and cardiac fibroblasts were lysed using RIPA protein extraction reagent with protease inhibitors (Beyotime, Shanghai, China). Then, equal amounts of proteins were run on 8–15% gels and blotted onto PVDF membranes. Subsequently, membranes were blocked with 5% non-fat dried milk in TBST and incubated with primary antibodies overnight at 4°C. The primary antibodies were Col III, Col I, MMP-2, TLR4, TGF-β (1:1000, Abcam, United States), Smad3, p-Smad3 (1:1000, Cell Signaling Technology, Inc.) and GAPDH (1:5000, Bioworld Technology, Inc.), as a loading control. After washing twice, membranes were incubated with appropriate HRP-conjugated secondary antibodies for 2 h at room temperature. Signals were detected using an ECL chromogenic substrate and quantified with densitometry by Quantity One software (Bio-Rad, Berkeley, CA, United States).

### Cell Viability Assays

Cell viability was measured using a Cell Counting Kit-8 (CCK8; Dojindo, WTS, Japan) to detect cell proliferation as described previously (Sun et al., 2017). Cells were seeded in 96-well plates at 5 × 10^3^ cells/well (100 μl). Cells were treated with different TMAO concentrations for 24 h, and then CCK8 (10 μl) was added to each well immediately for a 2 h incubation at 37°C. The absorbance was read at 570 nm (A570) using a microplate spectrophotometer.

### Immunofluorescence and Edu Staining

Cardiac fibroblasts were fixed in 4% paraformaldehyde, permeabilized with 0.2% Triton X-100 and then blocked with 10% TBST with goat serum. Subsequently, cells were incubated with α-SMA antibody (1:200, Abcam, United States) overnight in the dark. After washing twice, the cells were incubated with Cy3-labeled goat anti-rabbit lgG (H+L) (1:200, Abcam, United States) at room temperature in the dark for 2 h. DAPI was used for nuclear staining.

Edu staining was also performed to evaluate cell proliferation using Edu-Apollo^®^567 (RiboBio, Guangzhou, China) according to the manufacturer’s instructions. Briefly, after treatment with NLRP3 siRNA, Edu labeling solution was added to cardiac fibroblasts and incubated for 2 h. Subsequently, cardiac fibroblasts were fixed with 4% paraformaldehyde and 2 mg/ml glycine solution. After washing twice with PBS, cardiac fibroblasts were incubated with 1× Hoechst 3342 solution to label the cell nuclei. Finally, images were acquired using a Lecia fluorescence microscope (Leica, Germany).

### Cell Migration and Apoptosis Assays

Cell migration assays were assessed using a cell scratch assay and Transwell chambers (8-μm, Corning, United States) (Liu et al., 2018). Cardiac fibroblasts cultured in 6-well plates were treated with different TMAO concentrations and then subjected to a cell scratch assay with a 200 μl micropipette tip for 24 h following a protocol described previously (Liang et al., 2007). The width of the wound was measured by microscopy at 0 and 24 h. Cells were stained with 0.1% crystal violet at 24 h to better measure the width. Then, the migration rates (scratch width at 24 h/ scratch width at 0 h × 100%) were calculated from at least three separate experiments.

After treatment, the lower chamber was placed into a 24-well plate with medium containing 10% FBS. Then, 1 × 10^5^ cardiac fibroblasts suspended in serum-free DMEM were seeded into the upper chamber. After 24 h, cells that crossed the membrane were stained with 0.1% crystal violet. Finally, cells were counted from five random images of each chamber under a microscope.

Cell apoptosis was assessed with an Annexin Apoptosis Detection Kit APC (eBioscience, Inc.). Following double staining with 5 μl allophycocyanin (APC)-Annexin V and 10 μl propidium iodide (PI) for 10 min at room temperature in the dark, cells were examined by a FACScan (BD Biosciences, CA, United States) equipped with Cell Quest software (BD Biosciences).

### Oxidative Stress Assay

Intracellular oxidative stress was determined using dihydroethidium (DHE) according to the manufacturer’s instructions as indicated previously (Qin et al., 2018). After treatment, cardiac fibroblasts were incubated with 10 μM DHE (Beyotime, Shanghai, China) for 30 min at 37°C in the dark. The cells were observed under a fluorescence microscope and quantified using ImageJ software.

### Quantitative Real-Time PCR

Total RNA from mouse hearts was collected using TRIzol reagent (Invitrogen, Life Technology, United States). Total RNA was quantified using a NanoDrop 2000 spectrophotometer (NanoDrop, Wilmington, DE, United States), and 1 μg of total RNA from each sample was reverse transcribed into cDNA using HiScript II Q RT SuperMIx for qPCR (Vazyme, Nanjing, China). Quantitative real-time PCR (qRT-PCR) was performed in a Stepone plus Real-Time PCR system (Applied Biosystems) at 95°C for 30 s, followed by 45 cycles of 95°C for 10 s and 60°C for 30 s. The qRT-PCR data were normalized to the average levels of the housekeeping gene GAPDH. Relative mRNA expression levels were described as the 2-ΔΔCt value. The sequences of the primers used for PCR are as follows: Forward: 5′-AGCTTCAGGCAGGCAGTATC-3′, Reverse: 5′-TCATCTCGGAGCCTGTAGTG -3′ (Mouse IL-1β); Forward: 5′- AACTCCAGGCGGTGCCTATG -3′, Reverse: 5′- TCCAGCTGCTCCTCCACTTG -3′ (Mouse TNF-α); and Forward: 5′- GAGAAACCTGCCAAGTATGATGAC-3′, Reverse: 5′- AGAGTGGGAGTTGCTGTTGAAG -3′ (Mouse GAPDH).

### Caspase 1 Activity Assay

Caspase-1 activity was measured by using colorimetric assay (Beyotime, China) according to the manufacturer’s instructions (Luo et al., 2014; Qiu et al., 2017). This assay was based on the ability of caspase-1 to change acetyl-Tyr-Val-Ala-Asp p-nitroaniline (Ac-YVAD-pNA) into the yellow formazan product p-nitroaniline (pNA). 50 ug of total proteins from mouse hearts were incubated in a 96-well microtiter plate with 20 nmol Ac-YVAD-pNA overnight at 37°C. The absorbance was read at 405 nm (OD405) using a microplate spectrophotometer. Then the level of caspase-1 activation can be assessed by measuring the production of pNA in tested sample using a standard curve of pNA at 405 nm.

### Statistical Analysis

Data are presented as the mean ± SD of at least three independent experiments. The differences in data were analyzed by unpaired, two-tailed Student’s *t*-test for two groups or one-way analysis of variance (ANOVA) for multiple comparisons. SPSS 22.0 (IBM SPSS, Armonk, NY, United States) was used for statistical analyses, and statistical significance was indicated by *P* < 0.05.

## Results

### TMAO Exacerbated DOX-Induced Cardiac Dysfunction

**FIGURE 1:**
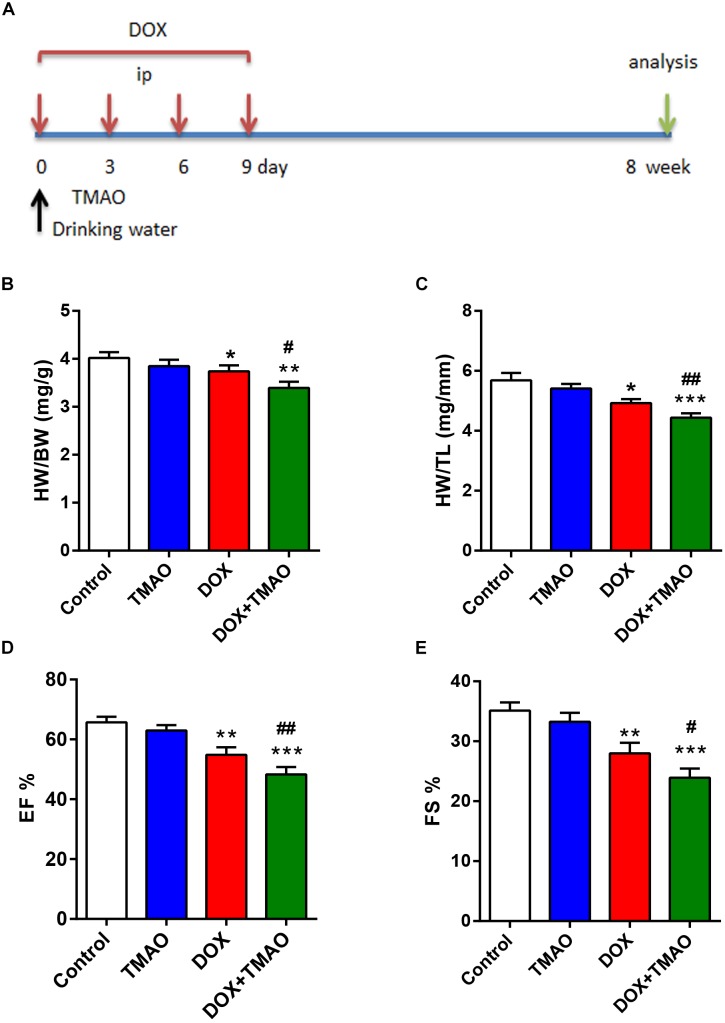
TMAO exacerbated doxorubicin (DOX)-induced cardiac dysfunction. **(A)** Schedule of the 8-week experiment. The mice received four doses of DOX (6 mg/kg) or PBS every 3 days by intraperitoneal injection (i.p.). Moreover, all mice were treated with or without TMAO (120 mg/kg, 1.60 mmol/l) in drinking water. **(B)** Heart weight/body weight (HW/BW) and **(C)** heart weight/tibial length (HW/TL) were analyzed. **(D)** EF and **(E)** FS of the mice were measured at 8 weeks. All data were analyzed at least three times. Mean ± SD. ^∗^*P* < 0.05, ^∗∗^*P* < 0.01, ^∗∗∗^*P* < 0.001 (vs. Control). ^#^*P* < 0.05, ^##^*P* < 0.01 (vs. DOX).

To analyze the effects of TMAO on cardiac fibrosis, we administered intraperitoneal injections of DOX to establish a murine cardiac fibrosis model. Then, the mice were given drinking water with or without TMAO for 8 weeks (Figure 1A). The mice treated with DOX showed significantly reduced relative heart weight, including HW/BW and HW/TL, which could be further reduced by additional treatment with TMAO (Figure 1B,C). An ultrasonic cardiogram was used to evaluate cardiac function in mice. After DOX treatment, all mice exhibited significant functional impairment in the left ventricular EF and FS. Importantly, poor cardiac function was completely deteriorated by TMAO (Figure 1D,E). However, no significant differences were seen in heart weight and cardiac parameters, including EF and FS, between control- and TMAO-treated normal mice, most likely due to the short-term effect and relatively low levels of TMAO. In addition, the similar results also were found in the other cardiac parameters including LVESD and LVEDD (Supplementary Figure S2) in Supplementary Materials.

### TMAO Aggravated DOX-Induced Cardiac Fibrosis

**FIGURE 2:**
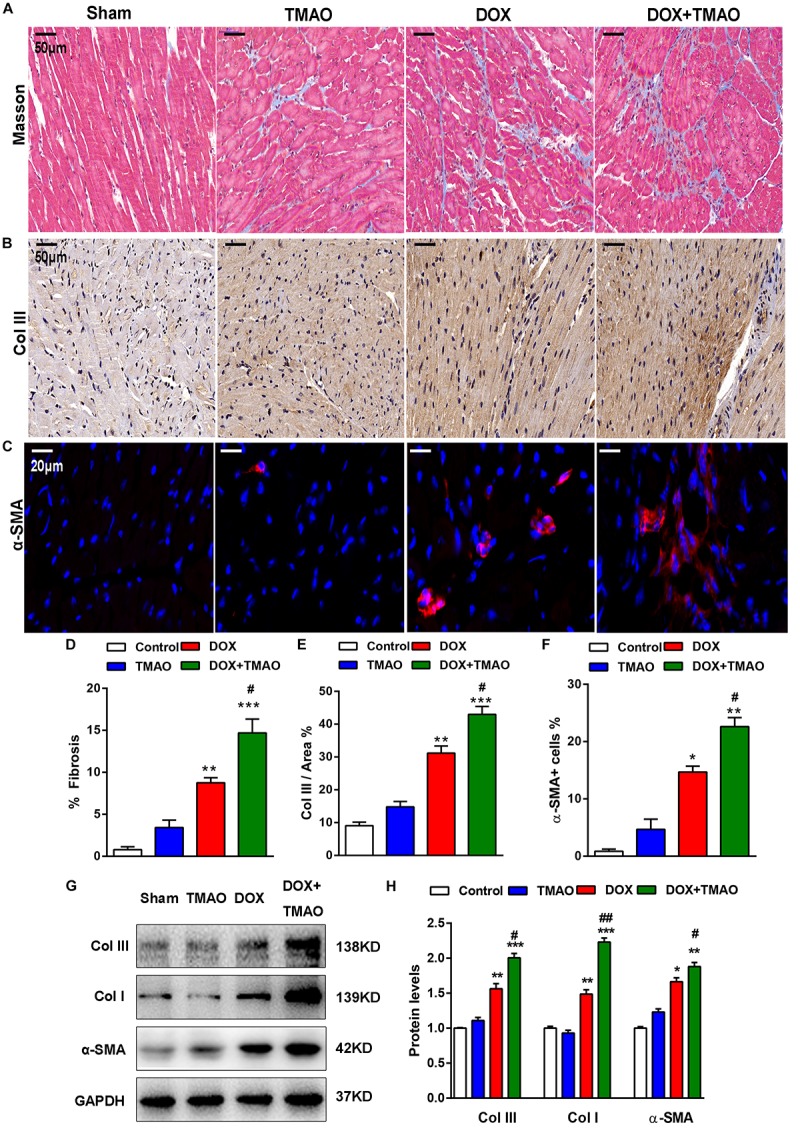
TMAO aggravated doxorubicin-induced cardiac fibrosis in mice. **(A)** Representative images of Masson’s staining and immunohistochemical staining for collagen type III (Col III) **(B)** and immunofluorescence staining for α-SMA (red) and DAPI (blue) in cardiac sections **(C)**. Scale bar = 50 μm. **(D)** Quantification of the percentage of fibrosis, Col III area **(E)** and α-SMA-positive cells **(F)**. **(G,H)** Collagen I (Col I), Col III, and α-SMA protein expression levels were analyzed by western blotting and quantified according to immunoblotting (the ratio of protein pixel density/GAPDH pixel density). All data were analyzed at least three times. Mean ± SD. ^∗^*P* < 0.05, ^∗∗^*P* < 0.01, ^∗∗∗^*P* < 0.001 (vs. Control). ^#^*P* < 0.05, ^##^*P* < 0.01 (vs. DOX).

Animals subjected to DOX treatment showed poor cardiac function and apparent cardiac fibrosis (Deng et al., 2007; Hang et al., 2017). Consistent with previous reports, we confirmed that DOX resulted in marked fibrosis and collagen deposition in mouse hearts by Masson and collagen type (Col) III staining (Figure 2A,B,D,E). These alterations were also observed in TMAO-treated mice, though to a lesser extent than those in the DOX-treated animals. Importantly, co-administration of TMAO and DOX resulted in more pronounced cardiac fibrosis than the administration of either alone in mice. Cardiac sections were stained for α-SMA, which is a common marker of cardiac fibroblasts. The proportion of α-SMA-positive cells in DOX-treated mouse hearts was markedly higher than that in control hearts and was further increased in combination with TMAO (Figure 2C,F). In addition, the expression levels of profibrotic factors were also detected by western blotting in all animals. DOX-treated animals showed increased expression levels of α-SMA, collagen type I, and collagen type III in the heart compared to untreated animals (Figure 2G,H). In agreement with these findings, TMAO was also able to slightly upregulate the levels of profibrotic factors; however, no significant differences were observed in normal TMAO-treated mouse hearts, although these factors were significantly upregulated by TMAO in DOX-treated mouse hearts.

### TMAO Induced Cardiac Fibroblast Proliferation, Migration, and Collagen Deposition

**FIGURE 3:**
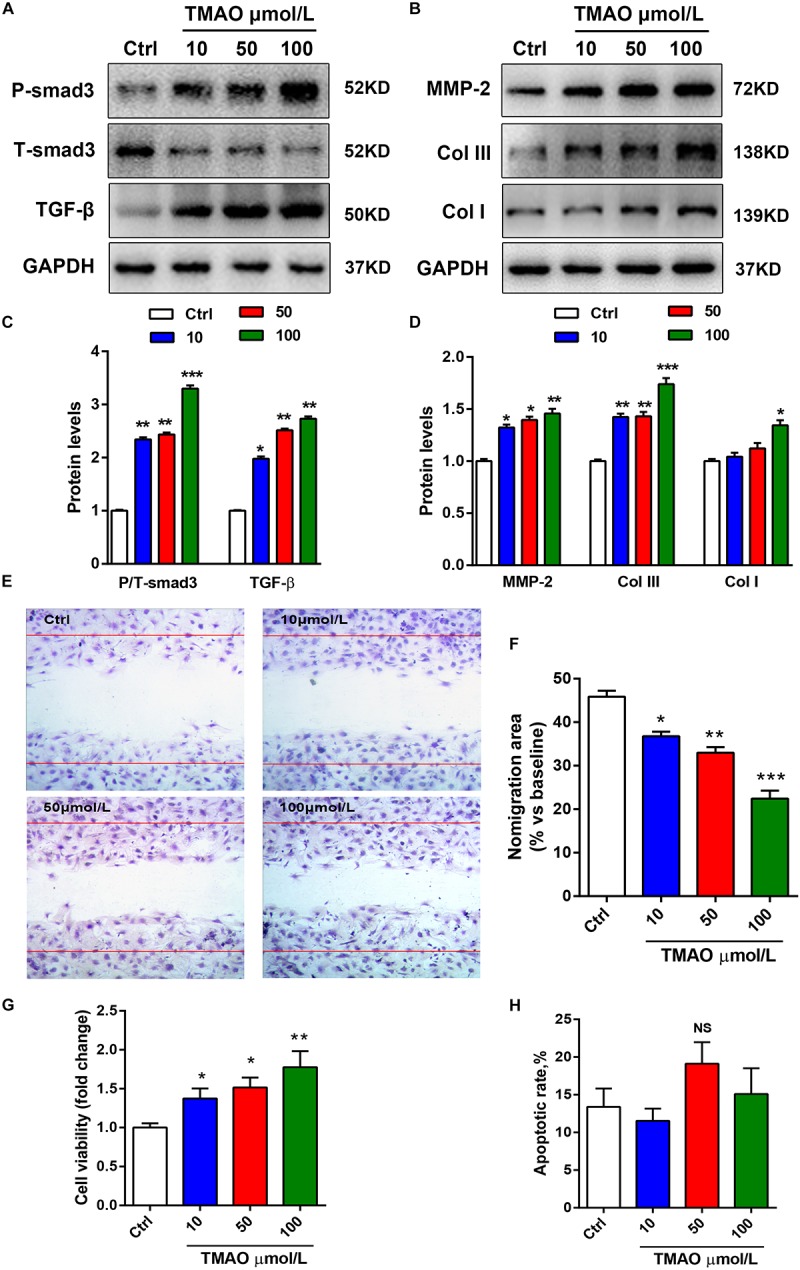
TMAO induced cardiac fibroblast proliferation, migration and collagen deposition in a dose-dependent manner. **(A)** P-Smad3, T-Smad3, TGF-β, MMP-2, Col I, and Col III **(B)** protein expression was analyzed by western blotting and quantified **(C,D)** according to immunoblotting (the ratio of protein pixel density/GAPDH pixel density) following different doses of TMAO exposure. **(E)** Scratch assays showing the migration ability of cardiac fibroblasts for 24 h under 200× magnification. **(F)** Quantification of the percentage of the migration area. **(G)** CCK-8 assay showing the proliferation of cardiac fibroblasts subjected to TMAO at different doses. **(H)** Quantification of the apoptotic rate of cardiac fibroblasts by an Annexin Apoptosis Detection Kit APC assays. All data were analyzed at least three times. Mean ± SD. ^∗^*P* < 0.05, ^∗∗^*P* < 0.01, ^∗∗∗^*P* < 0.001 (vs. Control).

To investigate the effects of TMAO on cardiac fibroblasts, we cultured cardiac fibroblasts with TMAO at several indicated concentrations for 24 h. TGF-β/Smad3 signaling plays a critical role in the development of fibrotic diseases. Therefore, we detected these profibrotic factor levels in TGF-β signaling by western blotting. Similar to the results from mouse hearts, TMAO increased the expression levels of profibrotic factors, including TGF-β and p-Smad3, in cultured cardiac fibroblasts in a dose-dependent manner (Figure 3A,C). A trend was also observed in collagen deposition-related protein expression, including Col I, Col III and MMP-2 levels (Figure 3B,D). Moreover, we performed scratch tests to detect the migration of cardiac fibroblasts in response to different doses of TMAO. The ratio of the migration area decreased in a dose-dependent manner, indicating that TMAO facilitated dose-dependent migration of cultured cardiac fibroblasts (Figure 3E,F). Furthermore, TMAO was also able to promote cardiac fibroblast proliferation in a dose-dependent manner as assessed by a CCK8 assay (Figure 3G). Interestingly, TMAO failed to induce cardiac fibroblast apoptosis, perhaps due to more proliferating cells and fewer apoptotic cells (Figure 3H). Overall, our results show that TMAO promotes cardiac fibroblast proliferation, migration and collagen secretion in a dose-dependent manner, indicating that TMAO contributes to cardiac fibrosis via regulating the function of cardiac fibroblasts.

### TMAO Induced NLRP3 Inflammasome Activation *in vivo* and *in vitro*

**FIGURE 4:**
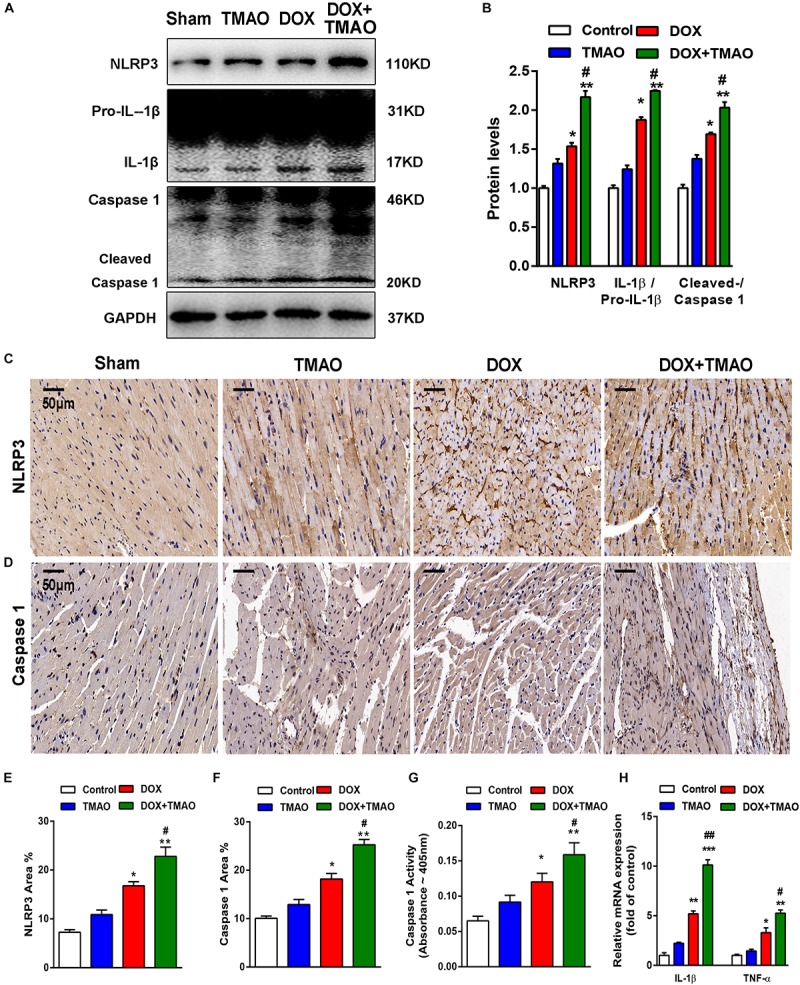
TMAO induced NLRP3 inflammasome activation *in vivo*. **(A)** NLRP3, pro-IL-1β, IL-1β, caspase 1 and cleaved caspase 1 protein expression was analyzed by western blotting and quantified **(B)** according to immunoblotting (the ratio of protein pixel density/GAPDH pixel density) after 8 weeks. **(C)** Representative images of immunohistochemical staining for NLRP3 and caspase 1 **(D)** in cardiac sections. Scale bar = 50 μm. **(E)** Quantification of the percentage of NLRP3- and caspase 1-positive areas **(F)**. **(G)** Caspase-1 activity was determined by caspase-1 activity assay. **(H)** QRT-PCR analysis of TNF-α and IL-1β mRNA expression levels in heart tissues. All data were analyzed at least three times. Mean ± SD. ^∗^*P* < 0.05, ^∗∗^*P* < 0.01, ^∗∗∗^*P* < 0.001 (vs. Control). ^#^*P* < 0.05, ^##^*P* < 0.01 (vs. DOX).

The NLRP3 inflammasome has been described as a key player in the development of cardiovascular disease. Previous investigations have revealed that TMAO could activate the NLRP3 inflammasome to mediate endothelial dysfunction and vascular inflammation (Boini et al., 2017; Chen M.L. et al., 2017). To explore the relationship between TMAO and the NLRP3 inflammasome in cardiac fibrosis, we detected the protein levels of the NLRP3 inflammasome complex, including NLRP3, caspase 1 and interleukin-1β (IL-1β), by western blotting. The levels of NLRP3 and IL-1β/pro- IL-1β were significantly increased in the DOX-treated groups compared to those in the control- and TMAO-treated groups, which were the highest in the TMAO+DOX group (Figure 4A,B). The ratio of cleaved caspase 1/caspase 1 was also higher in the TMAO+DOX-treated groups than either the TMAO- or DOX- treated group (Figure 4A,B). In addition, we detected the expression levels of NLRP3 and caspase 1 in mouse hearts by immunohistochemistry. Our results demonstrated that the expression of NLRP3 and caspase 1 could be observed in TMAO-treated hearts and that co-administration of TMAO and DOX further induced higher levels than either treatment alone (Figure 4C–F). Moreover, we also detect the activation levels of caspase 1 in mouse hearts by caspase1 activity assay kit. Consistent with the above results, the caspase 1 activity was significantly increased in in the DOX-treated groups compared to those in the control- and TMAO-treated groups but lower than TMAO+DOX group (Figure 4G). NLRP3 activation is involved in inflammation; thus, we analyzed the expression of inflammatory genes by quantitative real-time PCR. The data revealed that TMAO administration increased the expression of TNF-α and IL-1β but had no significant statistical difference (Figure 4H). Similarly, TMAO promoted a significant increase in inflammatory factor expression following DOX treatment (Figure 4H). The data supported that TMAO induced NLRP3 inflammasome activation in mouse hearts.

**FIGURE 5:**
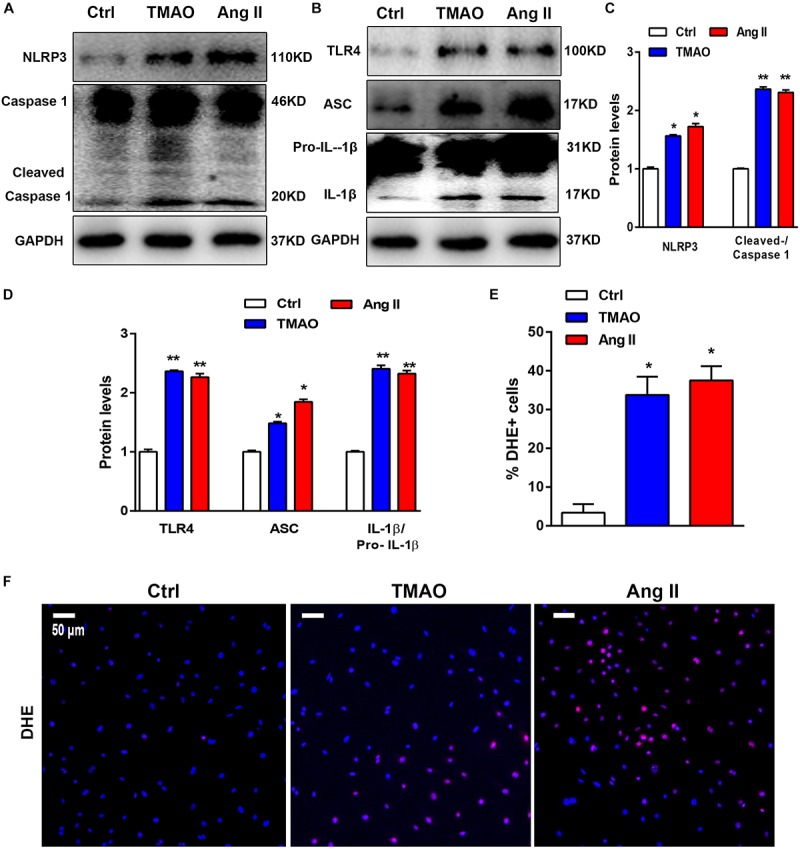
TMAO induced NLRP3 inflammasome activation *in vitro*. **(A,B)** NLRP3, pro-IL-1β,IL-1β, caspase-1, cleaved caspase-1, TLR4, and ASC protein expression levels were analyzed by western blotting and quantified **(C,D)** according to immunoblotting (the ratio of protein pixel density/GAPDH pixel density) after TMAO or Ang II treatment for 24 h. **(E,F)** Representative images and quantification of DHE staining (red) and DAPI (blue). Scale bar = 50 μm. All data were analyzed at least three times. Mean ± SD. ^∗^*P* < 0.05, ^∗∗^*P* < 0.01, ^∗∗∗^*P* < 0.001 (vs. Control).

We also evaluated the effect of TMAO on the NLRP3 inflammasome in cardiac fibroblasts using western blotting. To investigate the effects of TMAO on cardiac fibroblasts, we selected Ang II-treated cells as positive control. The results shown in Figure 5A–E indicated that the expression of NLRP3, cleaved caspase 1, pro-IL-1β, IL-1β and apoptosis-associated speck-like protein (ASC) significantly increased in the TMAO-treated cardiac fibroblasts, consistent with the observed increase in the AngII-treated group. In addition, TMAO also increased the expression of TLR4, which has been recognized as an important upstream molecule that regulates NLRP3 signaling (Gaidt et al., 2016; Figure 5B,D). Furthermore, oxidative stress was reported to be vital to NLRP3 inflammasome-mediated inflammation, so we detected reactive oxygen species (ROS) in cardiac fibroblasts by DHE staining. We found that TMAO increased the levels of ROS consistent with AngII treatment in cultural cardiac fibroblasts (Figure 5E,F). These results indicated that TMAO could activate the NLRP3 inflammasome and oxidative stress.

### TMAO Induced Cardiac Fibrosis via NLRP3 Activation

**FIGURE 6:**
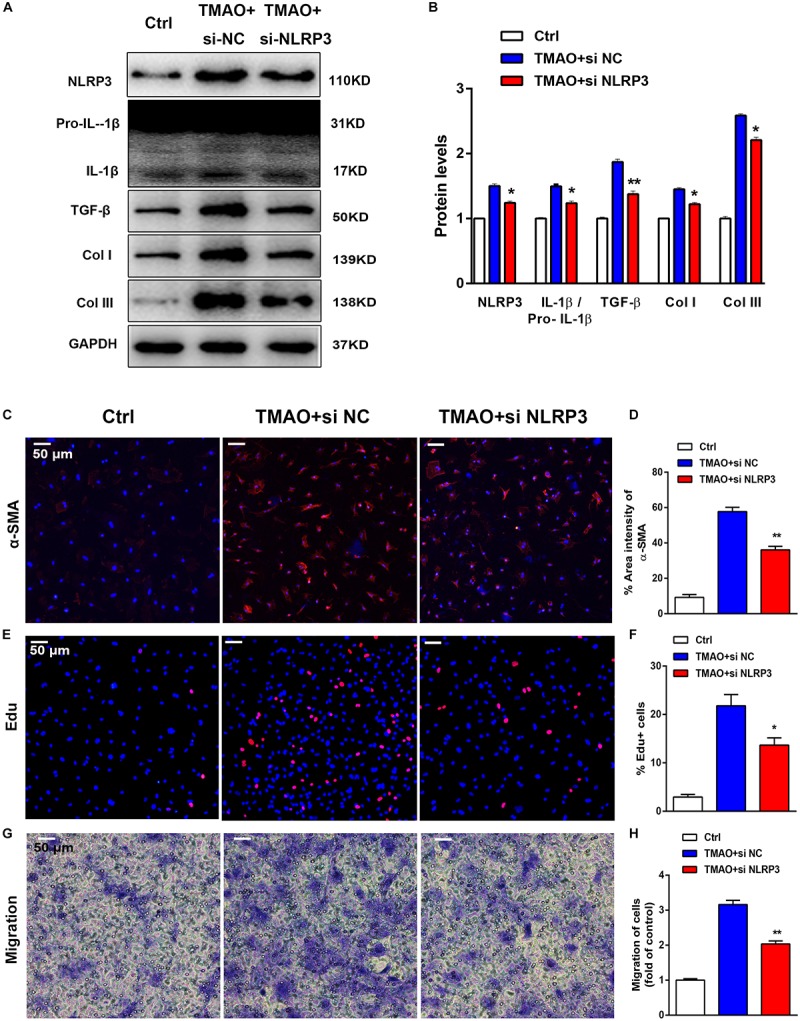
TMAO induced cardiac fibrosis via NLRP3 activation. Knockdown of NLRP3 with siRNA could reverse the effect of TMAO on cardiac fibroblasts. **(A)** NLRP3, pro-IL-1β, IL-1β, TGF-β, Col I and Col III protein expression levels were analyzed by western blotting and quantified **(B)** according to immunoblotting (the ratio of protein pixel density/GAPDH pixel density). **(C)** Representative images and quantification **(D)** of immunofluorescence staining for α-SMA (red) and DAPI (blue) in cardiac sections. **(E)** Edu staining showing cardiac fibroblast proliferation after 24 h of treatment, and quantification **(F)** of the percentage of Edu-positive cells (red). **(G)** Transwell assay showing the stained migrated cardiac fibroblasts after 24 h and calculation **(H)**. All scale bars = 50 μm. All data were analyzed at least three times. Mean ± SD. ^∗^*P* < 0.05, ^∗∗^*P* < 0.01, ^∗∗∗^*P* < 0.001 (vs. TMAO+si NC).

Considerable evidence has suggested that the NLRP3 inflammasome contributes to fibrosis in various diseases. TMAO activated NLRP3 inflammasome-initiated inflammation signaling, which led us to hypothesize that inhibition of NLRP3 with siRNA may protect against cardiac fibrosis induced by TMAO. Importantly, NLRP3 knockdown blunted the fibrotic effects of TMAO, including the attenuation of profibrotic factor levels and a reduction in the migration of cardiac fibroblasts. In our study, NLRP3 silencing abolished the increase in NLRP3, IL-1β/pro- IL-1β, Col I, TGF-β, and Col III levels induced by TMAO in cardiac fibroblasts (Figure 6A,B). Furthermore, TMAO increased the number of α-SMA-positive cells significantly compared with the control, and this effect was attenuated by NLRP3 silencing (Figure 6C,D). The Edu assay showed that TMAO increased cardiac fibroblast proliferation compared with the control condition and that this effect was significantly inhibited after NLRP3 silencing (Figure 6E,F). By counting migratory cardiac fibroblasts with a transwell assay, we found that TMAO increased the migration rate of cardiac fibroblasts, which was decreased by NLRP3 knockdown (Figure 6G,H). The above data demonstrated that NLRP3 inflammasome might be a possible mechanism in the development of cardiac fibrosis promoted by TMAO.

## Discussion

The present study aimed to explore the contributions of the role and physiological mechanism of NLRP3 inflammasome activation to cardiac fibrosis. Here, we observed that TMAO aggravated DOX-induced murine cardiac fibrosis and cardiac dysfunction. In addition, we found that TMAO induced cardiac fibroblast proliferation, migration and collagen synthesis. Furthermore, we showed that TMAO could activate the NLRP3 inflammasome as well as ROS production *in vivo* and *in vitro*. Finally, our study suggested that TMAO worsen cardiac fibrosis via NLRP3 inflammasome activation in DOX-treated mice.

In recent years, accumulating evidence has revealed that the gut microbiome plays a pivotal role in the onset and development of cardiovascular diseases (Crothers and Bry, 2018; Peng et al., 2018). TMAO, a major metabolite of the gut microbiome from nutrients, has been shown to be a risk factor that correlates with a higher mortality and worse prognosis of cardiovascular diseases, including atherosclerosis, atrial fibrillation and heart failure, in many clinical studies (Tang et al., 2014; Randrianarisoa et al., 2016; Yu et al., 2018). A growing body of research has demonstrated a strong association between TMAO and myocardial fibrosis. Therefore, inhibition of gut microbes or generation of TMAO may become a potential target for the prevention and treatment of cardiac fibrosis. In the present study, our results from heart weight, echocardiography, immunohistochemistry and western blotting revealed that TMAO might have no significant effect on heart weight and cardiac function, while it could induce mild cardiac fibrosis in normal mouse hearts. Indeed, a previous report has shown that dietary TMAO induced increased fibrosis in the kidney (Tang et al., 2015a). Cardiac fibrosis and collagen deposition were also observed in mice given water with supplemental TMAO, consistent with previous research (Organ et al., 2016). In addition, we demonstrated that TMAO exacerbated DOX-induced cardiac dysfunction and fibrosis in mouse hearts. Consistently, a recent study has reported that elevated circulating TMAO levels contribute to cardiac fibrosis and inflammation in Western diet-induced murine obesity (Chen K. et al., 2017). However, Huc et al. (2018) have found that increased dietary TMAO seems to reduce diastolic dysfunction and heart fibrosis in hypertensive rats. The discrepancy between Huc et al. (2018) and our findings may result from several factors, such as low doses and long-term treatment with TMAO in Huc’s study. Collectedly, these findings clarified that TMAO induced myocardial fibrosis and worsening cardiac function in DOX-treated mice.

Cardiac fibroblasts are known to play a vital role in heart fibrosis, which leads to cardiac dysfunction (Frangogiannis, 2018). Thus, we performed *in vitro* experiments to explore the effect of TMAO on cardiac fibroblasts, including proliferation, apoptosis, migration and collagen synthesis. We found that TMAO was able to induce the proliferation, migration and collagen secretion of cardiac fibroblasts in a dose-dependent manner but had no effect on apoptosis. Perhaps because TMAO induced proliferating cardiac fibroblasts to a greater extent than it does apoptotic cells, no significant increase in apoptosis rates was observed. These data demonstrated that treatment with TMAO contributed to cardiac dysfunction in mice by modulating the functions of cardiac fibroblasts, which are associated with increased proliferation, migration and collagen secretion. However, TMAO had no significant effect on normal mouse hearts, most likely due to the low doses, smaller number of animals and short-term treatment, which is consistent with the results of previous studies (Chang et al., 2015; Chen K. et al., 2017).

The NLRP3 inflammasome, a multiprotein complex including NLRP3, caspase-1, IL-1β and ASC, has been widely shown to be an essential step in cardiovascular disease and is linked to atrial fibrillation, atherosclerosis, diabetic cardiomyopathy and cardiac remodeling (Suetomi et al., 2018; Yao et al., 2018; Zhang et al., 2018). When activated by diverse stimuli, including ROS, pathogen-associated molecular patterns (PAMPs) and calcium influx, NLRP3 forms a protein complex with the adapter molecule ASC and the effector protein caspase-1, regulating the activation of the protease caspase-1 and cytokine IL-1β. Moreover, NLRP3 activated by Ca^2+^ may trigger mitochondrial damage, leading to an increase in ROS production (Pavillard et al., 2018; Yang et al., 2018). In some vivo studies, siRNA-mediated knockdown or genetic deletion of NLRP3 experiments prevented pathological cardiac remodeling and decreased ischemic damage and cardiac fibrosis under cardiac injury conditions (Sandanger et al., 2013; Zhang et al., 2014; Bugyei-Twum et al., 2016). In accord with these findings, we also showed that TMAO activated NLRP3 inflammasome signaling in cultured cardiac fibroblasts, which was consistent with the increase in fibrotic factor levels induced by TMAO. Additionally, the levels of ROS in cardiac fibroblasts were found to be elevated after TMAO or AngII treatment. Furthermore, we blocked the expression of NLRP3 by siRNA to explore the association between NLRP3 and fibrosis under TMAO treatment. Importantly, TGF-β/Smad3 signaling has been implicated as a principal mediator of the fibrotic response associated with inflammation and tissue injury and could be regulated by NLRP3 in fibrotic disease as described in previous studies (Khalil et al., 2017; Tian et al., 2017). Moreover, TLR4, which is upstream of NLRP3 and TGF-β signaling, plays a role in the fibrotic response. Under the pathological conditions, inactive NLRP3 was generated in the cytoplasm by TLR4 in response to pathogen-associated molecular patterns or danger-associated molecular patterns (DAMPs). Thus, we speculated that TMAO might induce heart tissue injury and produce DAMPs, and then activate the TLR4-NLRP3-TGF-β signaling pathway, which is associated with an increase in ROS production, inflammatory cytokines and collagen deposition, finally leading to severe cardiac fibrosis and cardiac dysfunction, providing a potential novel therapy approach.

At present, some mechanisms of the effect of TMAO in cardiovascular diseases have been proposed. In the pathological process of atherosclerosis, TMAO accelerated endothelial dysfunction through the activation of PKC/NF-κB/VCAM-1 (Ma et al., 2017). TMAO has also been found to reverse GFP-PDS-mediated endoplasmic reticulum perturbation, contributing to endoplasmic reticulum balance (Shepshelovich et al., 2005). Previous studies revealed that TMAO may exacerbate cardiac fibrosis and inflammation induced by high glucose levels via upregulating the proinflammatory cytokines TNF-α and IL-1β (Chen K. et al., 2017). Li et al. (2018) showed that gut microbe-derived metabolite TMAO induced transverse aortic constriction (TAC)-induced cardiac hypertrophy and fibrosis involving Smad3 signaling. The NLRP3 inflammasome has been implicated in cardiac inflammation and fibrosis. However, the biological role of the NLRP3 inflammasome in TMAO-induced cardiac fibrosis is still unclear. Our results indicated for the first time that TMAO induced cardiac fibrosis through the activation of the NLRP3 inflammasome *in vivo* and *in vitro*, suggesting that the NLRP3 inflammasome plays an important role in cardiac fibrosis after TMAO treatment. In addition, we showed that inhibition of NLRP3 could reverse TMAO-mediated cardiac fibrosis, indicating a potential target for the treatment of cardiac fibrosis.

Some limitations of this study should be pointed out. Gut microbiota analysis was not performed, and we could not analyze the gut microbiota composition and circulating TMAO levels in our animal models. Previous reports have shown that additional dietary TMAO could increase circulating TMAO levels in mouse plasma (Organ et al., 2016; Chen K. et al., 2017). Thus, further studies including gut microbiota analysis are needed. In addition, TMAO has been implicated in inflammation, including myocardial infarction, atherosclerosis, and diabetes mellitus (Janeiro et al., 2018; Kasselman et al., 2018). Apart from the NLRP3 inflammasome and ROS in our study, more inflammatory processes, such as NF-κB, STAT3, and MAPK signaling, could be further verified. Finally, the mechanism underlying the NLRP3 inflammasome and TGF-β-mediated fibrosis in cardiac fibroblasts has not yet been identified. Thus, more studies are needed.

In conclusion, our findings showed that TMAO could aggravate cardiac dysfunction and heart weight in DOX-treated mice. In addition, we showed that TMAO promoted myocardial fibrosis via TGF-β signaling *in vitro* and *in vivo* and that the activation of the NLRP3 inflammasome played a crucial role in TMAO-induced cardiac fibrosis. Therefore, our results for the first time help to clarify the association between TMAO and the NLRP3 inflammasome, suggesting a new potential target for preventing the progression of cardiac fibrosis.

## Data Availability

The raw data supporting the conclusion of this manuscript will be made available by the authors, without undue reservation, to any qualified researcher.

## Ethics Statement

All mouse protocols were approved by the Institutional Ethics Committee of the Nanjing Drum Tower Hospital and carried out according to the guidelines of the United States Department of Health (NIH Publication No. 85-23, revised 1996) for the use and care of laboratory animals.

## Author Contributions

XL and JG conceived and designed the experiments. All authors performed the experiments, analyzed the data, and wrote the manuscript.

## Conflict of Interest Statement

The authors declare that the research was conducted in the absence of any commercial or financial relationships that could be construed as a potential conflict of interest.
