# Supplementary material for: Trimethylamine N-Oxide Exacerbates Cardiac Fibrosis via Activating the NLRP3 Inflammasome
Source: Front Physiol. 2019 Jul 9;10:866. doi: 10.3389/fphys.2019.00866 (PMC6634262; doi:10.3389/fphys.2019.00866)
Supplement: Supplementary file 1 [file Presentation_1.pdf]

Supplemental figures

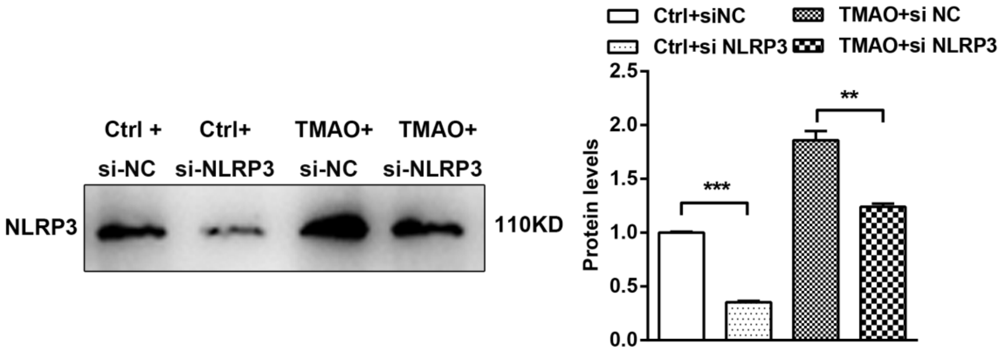

Fig.S1. NLRP3 silencing with siRNA could inhibit the expression levels of NLRP3 in cardiac fibroblasts. NLRP3 protein expression levels were analyzed by western blotting and quantified according to immunoblotting (the ratio of protein pixel density/GAPDH pixel density). All data were analyzed at least 3 times. Mean ± SD. ** P<0.01, *** P<0.001.

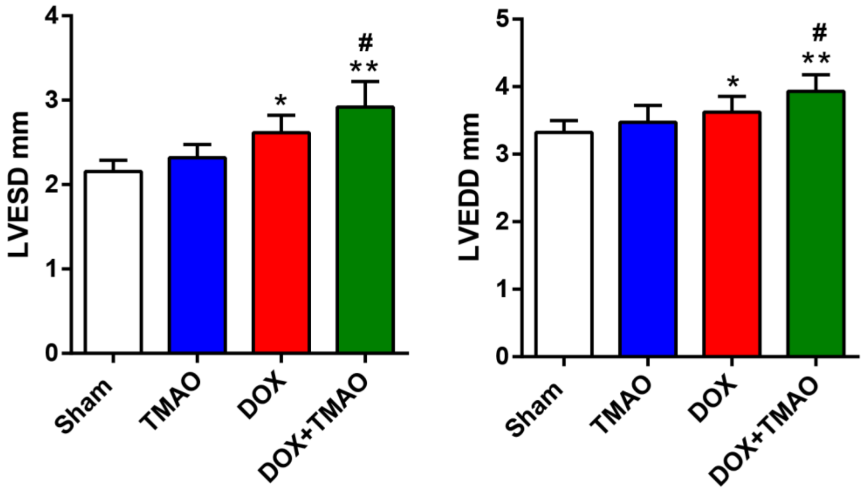

Fig.S2.LVESD and LVESD of the mice were measured at 8 weeks. Mean ± SD. * P < 0.05, ** P<0.01, (versus Control). # P < 0.05 (versus DOX).
